# Different levels of glyphosate-resistant *Lolium rigidum* L. among major crops in southern Spain and France

**DOI:** 10.1038/s41598-017-13384-2

**Published:** 2017-10-13

**Authors:** Pablo Tomás Fernández-Moreno, Ilias Travlos, Ivo Brants, Rafael De Prado

**Affiliations:** 1 Department of Agricultural Chemistry and Edaphology, University of Cordoba, 14071 Cordoba, Spain; 2 Faculty of Crop Science, Agricultural University of Athens, Athens, Greece; 3 Monsanto Europe SA, 1150 Brussels, Belgium

## Abstract

Herbicides are the most effective tools for controlling almost 99% of weeds. However, herbicide resistance is a primary concern in modern agriculture. The characterization in new areas and elucidation of the mechanisms of resistance are of vital importance in maintaining the sustainability of herbicides, including glyphosate. Nine populations of *Lolium rigidum*, showing different response patterns, were characterized as being glyphosate resistant (GR). The wide range of values in fresh weight reduction, survival, shikimic acid and EPSPS enzyme activity indicates a different or a combination resistance mechanism. The Line-3 population resulted in minimum reduction of fresh weight and survival values with respect to the glyphosate-susceptible (GS) population, showing 16.05- and 17.90-fold higher values, respectively. There were significant differences in the ^14^C-glyphosate translocation between GR and GS populations. Moreover, there were differences among the nine GR populations, but they exhibited a reduction in the remaining glyphosate translocation in the treated leaf. The EPSPS gene sequence revealed a Pro-106-Ser substitution in four populations, which could be characterized as being GR with non-target-site and target-site resistance mechanisms. This complexity of several resistance mechanisms makes it necessary to develop long-term integrated weed management strategies to limit further resistance dispersal.

## Introduction

Non-controlled weeds cause approximately 34% loss in crop yields worldwide^1^. Herbicides are the most effective weed control tools, and control approximately 99% of weeds^2^. Herbicide resistance is due to the evolution of weed adaptation following selection pressure from repeated herbicide applications and is undoubtedly a major concern in modern agriculture^3^. Glyphosate is one of the most widely used herbicides, although it also belongs to a class of herbicides with cases of resistance reported in many weed species^4^. To date, the number of glyphosate-resistant weeds has grown to 37 species worldwide^5^. Although glyphosate resistance has reached a peak level, it has been controlled by the adoption of best management practices. Glyphosate is still considered a very efficient herbicide, especially under an herbicide-rotation regime where herbicides with different modes of action are used for reducing the glyphosate selection pressure^6^.

This globally important herbicide (glyphosate) inhibits the nuclear-encoded enzyme 5-enolpyruvylshikimate-3-phosphate synthase (EPSPS) (EC 2.5.1.19), which catalyses the reaction of shikimate-3-phosphate and phosphoenolpyruvate (PEP) to form 5-enolpyruvylshikimate-3-phosphate, which is an important step in the biosynthesis of aromatic amino acids in plants^7^. Resistance has been documented for the modes of action of all major known herbicides; however, no new modes of action have been commercialized since the 1980’s^8^. Glyphosate-resistant weed survival is due to different types of resistance mechanisms classified as target site (TSR) and non-target site resistance (NTSR)^9,10^. The TSR mechanism is endowed by alterations in the gene encoding the herbicide target protein, causing an increased expression of the target protein or structural changes in the herbicide binding site^11,12^. The TSR mechanisms include a target-site mutation (Pro-106 substitution), which was first detected in *Eleusine indica* ^13^ and which, over the years, has been identified in other weeds^14–16^, genomic EPSPS duplication^17^, and overexpression of EPSPS^18^. Moreover, a double mutation in *E. indica* and *Bidens pilosa* was found in the Thr-102-Ile position followed by Pro-106-Ser^10,11^. While a single target-site mutation in the EPSPS gene seems to confer low levels of resistance to glyphosate in the order of two- to fourfold, the double mutation greatly increases resistance levels^10–12^.

The NTSR mechanism resulted from reduced absorption and/or translocation, increased vacuolar sequestration, and metabolism to non-toxic compounds, causing a lesser glyphosate transport via the phloem to the EPSPS^2,19^. NTSR has been described as the most common mechanism of resistance to glyphosate^9,20^. The NTSR mechanism can confer unpredictable resistance to herbicides following different modes of action^21,22^. Similar to TSR, several glyphosate-resistant weeds have been characterized, with NTSR being the mechanism involved in the resistance^23–28^.

*Lolium* spp. is an important genus of grass weeds worldwide, with three major species: *Lolium rigidum*, *L. multiflorum*, and *L. perenne* ^29^. These species are principally weeds in perennial crop spheres such as olive groves, vineyards or orchards, and cereal crops. In France and Spain, these grass weeds are widely distributed among the major crops^4,15^. *L. rigidum* is one of the most difficult to control, has the tendency to evolve resistance to herbicides and is reportedly resistant to eleven different herbicide action sites^5^. This situation reduces the options of diversification with alternative herbicides for an optimal management of this grass weed.

The main objectives of the present study were as follows: a) the screening of different *L. rigidum* populations from France and Spain as well as the determination of their resistance potential to glyphosate, and b) the evaluation of the mechanisms involved in their resistance to glyphosate.

## Results

### Population screening

**Figure 1:**
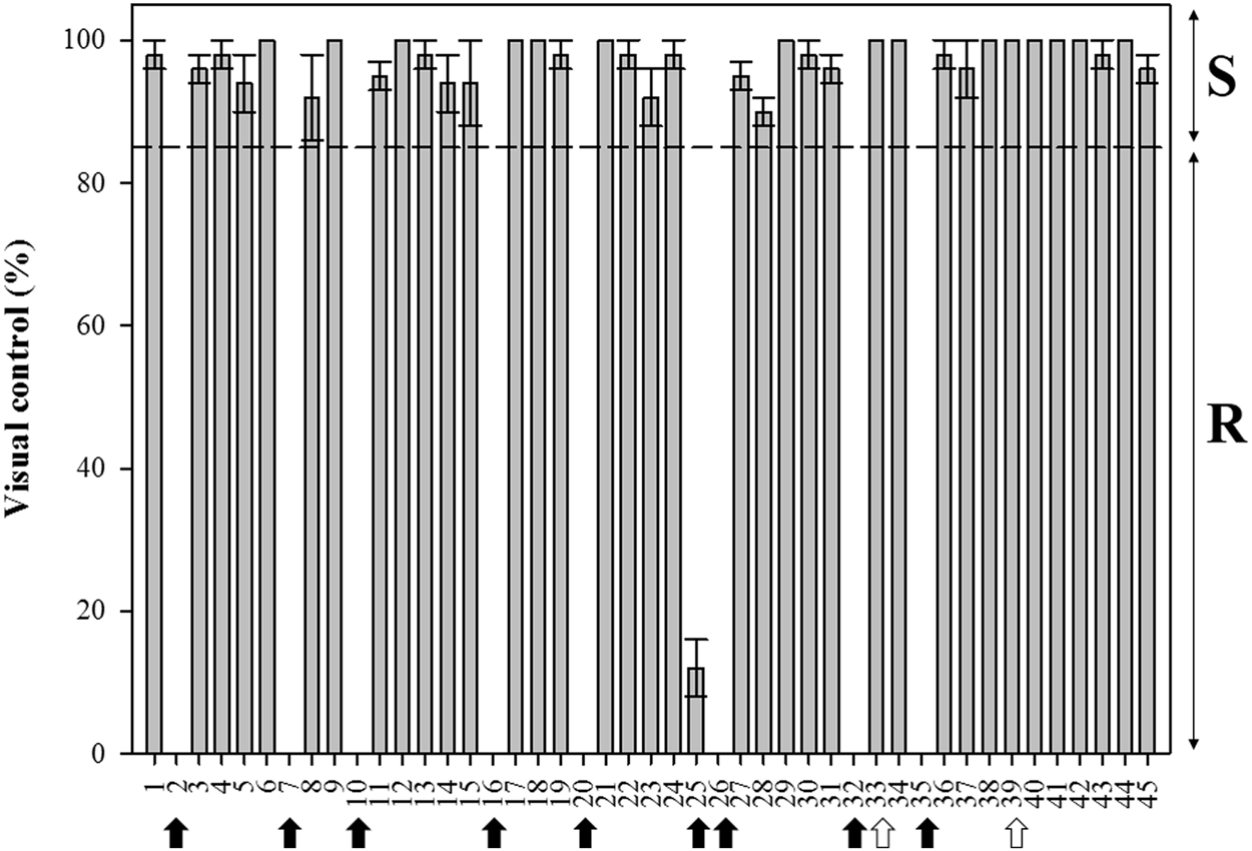
Visual control (%) of populations collected from different areas of Spain and France. A visual evaluation ranging from a scale of 0 to 100 was performed to determine the glyphosate resistance at 21 days after application (DAT). A score of 100 meant total glyphosate control, and 0 meant no control. Populations with a score of 85 or over were characterized as being GS, while populations with a lower score were characterized as being GR. Black arrows mean the 9 GR populations, and white arrows mean the 2 GS populations used for the following assays. Values represent mean (*n* = 50) and vertical bars represent ± standard errors.

Nine populations exhibited resistance (GR) to glyphosate and, survived the recommended field dose (more than 1080 g ae ha^−1^). Almost the GR populations with their 50 plants treated did not exhibit any symptoms of damage caused by the herbicide application (0 score in these populations), but only the Wheat-A population showed a little damage, in which 6 to 50 plants were controlled (Fig. 1). The remaining populations characterized as susceptible (GS) to glyphosate exhibited a score ranging from 95–100, with non-significant differences among the GS-populations (Fig. 1). Optimal efficacy was observed on these GS-populations, without any regrowth symptoms or possible plant survival after glyphosate application. We obtained the historical field application records for only some populations out of the 45 populations; thus, we collected seeds from nearby areas to obtain more information. In most cases, farmers applied glyphosate in advanced growth stages (beginning of flowering stage), and without alternate with other herbicide action modes for a long time ago. In perennial crops, the use of oxyfluorfen has been increased due to the poor effectiveness of glyphosate. The population numbers 7 to 11 were from nearby fields (Supplementary Table S1), two of which were GR (Alamo and Line-3). If the dispersal of GR-seeds is not controlled, they could spread and become established in the susceptible areas. This situation arose for numbers 25 (Wheat-A) and 26 (Wheat-B). The Wheat-B population, from a wheat field, which had been exposed to glyphosate for several years after wheat harvest and before in order to control the weeds and prepare the soil surface for the next crop, usually sunflower or corn (rotational weed management). Conversely, the Wheat-A population, which was from an olive grove near this cereal crop, never presented glyphosate performance problems; however, numbers 27 and 28, from the same farm as number 26, showed susceptibility. Therefore, the resistance of Wheat-A was possibly due to the dispersal seeds.

### *Lolium* weed species characterization using molecular markers

**Figure 2:**
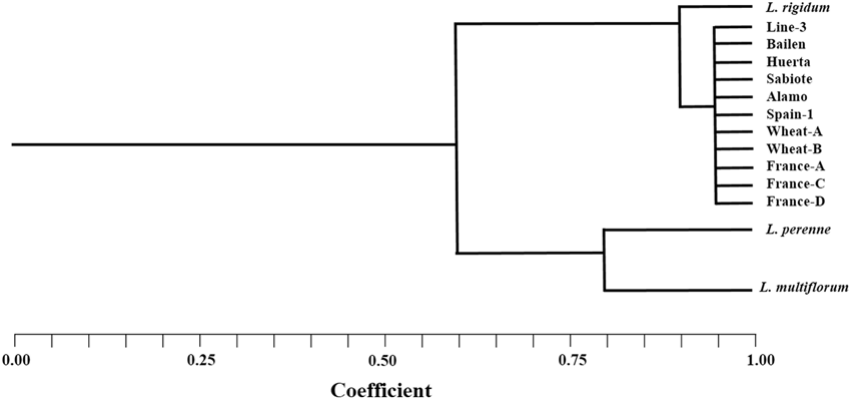
Dendogram of the genetic similarities among *Lolium* species after UPGMA analysis performed with AFLP marker data. Twelve plants of each putative population were used for molecular analysis.

Jaccard’s similarity indices were calculated for all the populations examined, and the clustering was represented by a dendrogram. In the UPGMA dendrogram, two main clusters converged at a 60% similarity level. The first cluster comprised an*L*. *rigidum* control population with all the populations assayed in this study. The second cluster was formed by *L. multiflorum* and *L. perenne* control populations. According to these results, the *Lolium* species (infesting crops from southern Spain and France) used in this study correspondedto *L. rigidum* (Fig. 2).This assay helped us to determine that all populations, both GR and GS, that were to be studied belonged to the same species. Otherwise, we would not know if we would be comparing between populations of different species.

### Dose-response assays

The nine GR populations found in the screening assay were studied by means of dose-response experiments. Moreover, two GS populations were studied, which exhibited GR_50_ values of 91.07 and 73.99 g ae ha^−1^; and LD_50_ values of 232.18 and 204.72 g ae ha^−1^ for Spain-1 and France-C, respectively. There were no differences found between France-C and Spain-1 (P = 0.289). Therefore, each population was compared with France-C to obtain the Resistance Index (RI).

**Figure 3:**
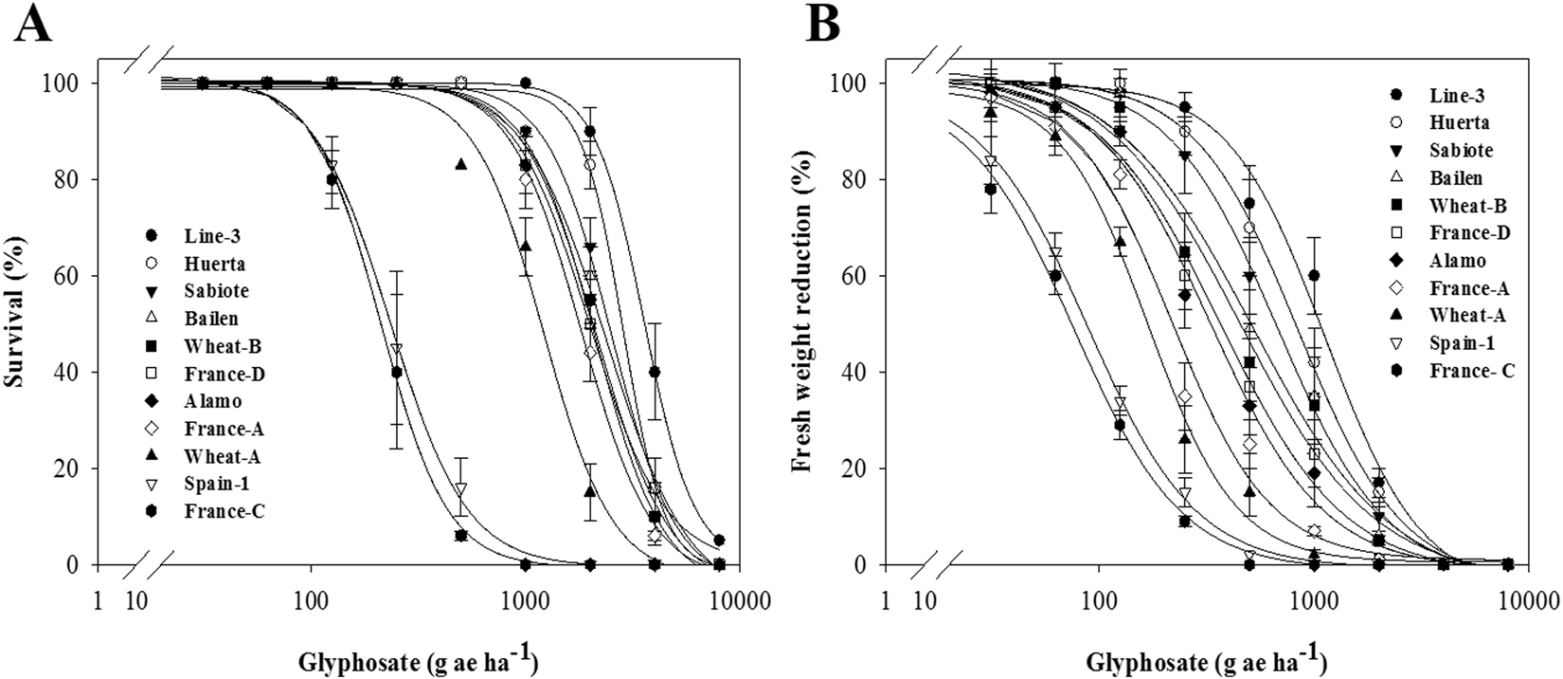
Glyphosate dose-response on (**A**) plant survival and (**B**) above-ground fresh weight expressed as percentage of the mean untreated control of the *Lolium rigidum* populations. Values represent mean (*n* = 10) and vertical bars represent ± standard errors.

Diverse results were observed in different populations (Fig. 3A,B). The Line-3 population obtained the maximum GR_50_ and LD_50_ values with respect to France-C, showing 16.05- and 17.90-fold higher resistance. The Huerta, Sabiote, Bailen, and Wheat-B populations showed lower LD_50_ values with respect to Line-3, but their values were higher than 2160 g ae ha^−1^ (6 L ha^−1^, 360 g ae L^−1^). The France-D, Alamo, France-A, and Wheat-A populations showed LD_50_ values of approximately 2047.38 and 1138.63 g ae ha^−1^ (Table 1). These nine populations were characterized as GR because they survived the recommended field dose (3 L ha^−1^, 360 g ae L^−1^).

**Table 1:** Parameters of the log-logistic equations used to calculate the glyphosate rates required for 50% reduction survival (LD_50_) and fresh weight (GR_50_), expressed as percentage of the mean untreated control of the *Lolium rigidum* populations.

Population	d	b	LD_50_ (g ae ha^−1^)	(95% CI)		RI	*p-value*
				Upper	Lower		
Line-3	100.10	3.77	3664.83	3705.66	3624.00	17.90	<0.0001
Huerta	98.81	4.52	2893.28	2929.21	2857.35	14.13	<0.0001
Sabiote	99.84	2.89	2645.71	2748.12	2543.30	12.92	<0.0001
Bailen	100.24	2.26	2314.93	2407.67	2222.19	11.30	<0.0001
Wheat-B	100.14	2.53	2183.44	2257.05	2109.83	10.66	<0.0001
France-D	100.44	2.47	2047.38	2090.65	2004.11	10.00	<0.0001
Álamo	100.13	2.73	1983.73	2052.80	1914.66	9.68	<0.0001
France-A	100.40	2.62	1710.62	1793.71	1627.53	8.35	<0.0001
Wheat-A	99.34	2.70	1138.63	1193.64	1083.62	5.56	0.003
Spain-1	101.44	2.42	232.18	248.93	215.43	1.13	0.289
France-C	100.59	2.79	204.72	228.20	181.24	—	—
**Population**	**d**	**b**	**GR**_**50**_ **(g ae ha** ^**−1**^ **)**	**(95% CI)**		**RI**	***p-value***
				**Upper**	**Lower**		
Line-3	99.92	1.93	1188.06	1225.73	1150.39	16.05	<0.0001
Huerta	100.91	1.79	813.60	834.57	792.63	10.49	<0.0001
Sabiote	101.20	1.71	625.39	647.31	603.47	8.45	<0.0001
Bailen	103.36	1.34	515.34	538.22	492.46	6.96	0.0006
Wheat-B	103.28	1.45	465.51	468.18	462.84	6.29	0.0002
France-D	101.94	1.50	366.06	377.27	354.85	4.94	0.0001
Álamo	101.19	1.64	339.45	356.78	322.12	4.58	0.001
France-A	100.42	1.89	202.91	219.34	186.48	2.78	0.009
Wheat-A	98.90	2.12	154.53	164.81	144.25	2.08	0.008
Spain-1	99.53	1.72	91.07	111.74	70.40	1.23	0.743
France-C	98.72	1.73	73.99	79.36	68.62	—	—

CI values are the 95% confidence intervals (*n* = 10). RI (Resistance Indices) = GR_50_, or LD_50_ (Resistant)/GR_50_, or LD_50_ (Susceptible). d is the coefficient corresponding to the upper asymptote, b is the slope of the line.

These results could be divided into two resistance levels. The first one was characterized by higher GR-populations and comprised Line-3, Huerta, Sabiote, Bailen, and Wheat-B. The second one is comprised France-D, Alamo, France-A and Wheat-A, which have lower resistance levels than the above but higher resistance levels than the field dose (Table 1). The differences in the results may be attributed to the different mechanisms involved and the cumulative effect of the different mechanisms. Therefore, we studied these mechanisms to identify the cause of these different results.

### Shikimic acid accumulation

**Figure 4:**
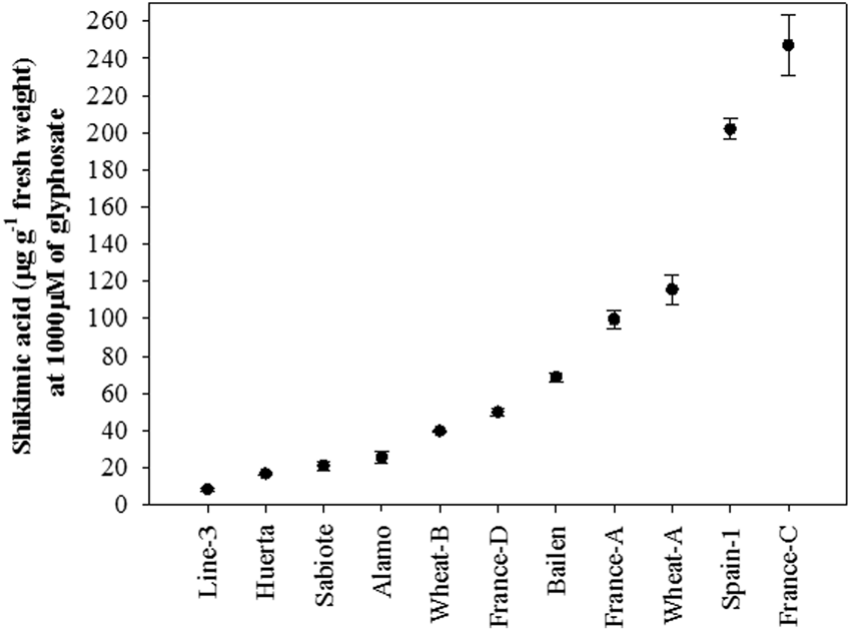
Shikimic acid accumulation in leaf segments (50 mg [5 mm diameter] from a pool of 15 plants per population) of *Lolium rigidum* populations. Vertical bars are ± standard errors of the mean.

Figure 4 indicates the shikimic acid accumulation at 1000 µM of glyphosate. As observed in the dose-response assays, the two S-populations (France-C and Spain-1) accumulated the highest amounts of shikimic acid. France-C presented 247.07 µg shikimic acid g^−1^ fresh weight, 30.13-fold higher with respect to Line-3, the population with the highest GR rate.

### ^14^C-glyphosate absorption translocation and visualization

In this study, the ^14^C-glyphosate recovery indifferent populations was approximately 90–95%. Different ^14^C-glyphosate absorption and translocation values were observed at 96 h after treatment (HAT), and the maximum amounts were observed at that time (Table 2). The results ranged from 32% (lower absorption) for the Line-3 population to 85% (higher absorption) for the France-C population. Also, there were significant differences between populations. The remaining populations had intermediate values compared with the values mentioned above. From lower to higher absorption results, the populations were as follow: Line-3, France-D, Huerta, Spain-1, Sabiote, France-A, Wheat-A, Wheat-B, Alamo, Bailen, and France-C. The Spain-1 (GS population) did not exhibit differences with other GR populations as Sabiote, Alamo, Wheat-B, France-A, and Wheat-A. Moreover, France-C (GS population) showed the similar results to Bailen. Reduced absorption was not a potential mechanism involved in the resistance of these populations (Table 2).

**Table 2:** ^14^C glyphosate absorption and translocation in *Lolium rigidum* populations at 96 h after treatment (HAT).

Population	Absorption^a^ (p < 0.0001, DF = 10, n = 55)	Translocation^b^		
		Treated leaf (p < 0.0001, DF = 10, n = 55)	Shoot tissue (p < 0.0001, DF = 10, n = 55)	Roots (p < 0.0001, DF = 10, n = 55)
Line-3	32.49 ± 10.29 D	63.43 ± 10.71 ABC	21.68 ± 2.93 C	14.88 ± 2.88 E
Huerta	50.57 ± 9.08 C	66.82 ± 12.03 AB	16.19 ± 4.64 CD	16.98 ± 7.68 E
Sabiote	58.73 ± 9.11 BC	50.79 ± 3.35 EF	33.05 ± 9.35 B	16.14 ± 7.38 E
Bailen	87.87 ± 5.44 A	59.71 ± 2.58 BCD	32.81 ± 7.08 B	7.47 ± 1.23 F
Wheat-B	64.35 ± 10.01 B	23.80 ± 2.08 H	57.11 ± 4.25 A	19.07 ± 3.01 DE
France-D	37.28 ± 2.70 D	56.74 ± 10.50 CDE	15.55 ± 3.58 CD	27.69 ± 4.14 C
Álamo	68.24 ± 4.27 B	67.94 ± 12.22 A	13.38 ± 3.76 CD	18.66 ± 3.87 E
France-A	59.38 ± 17.88 BC	55.09 ± 6.17 DE	19.25 ± 1.27 CD	24.94 ± 5.14 CD
Wheat-A	61.44 ± 9.20 BC	45.93 ± 3.98 FG	19.76 ± 10.85 CD	34.30 ± 3.05 B
Spain-1	58.24 ± 9.64 BC	28.72 ± 1.09 H	22.09 ± 4.38 C	49.17 ± 6.29 A
France-C	84.77 ± 2.53 A	39.75 ± 3.79 G	11.55 ± 2.04 D	48.68 ± 2.54 A

Mean value (*n* = 5) ± standard error of the mean. Means on a same column followed by the same letter were not significantly different at α = 0.05. Differences between means were separated using the Tukey HSD test. ^a^Percent of applied label. ^b^Percent of absorbed label.

**Figure 5:**
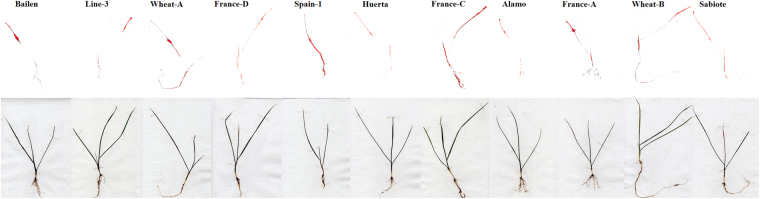
^14^C-glyphosate visualization for the different *Lolium rigidum* populations at 96 HAT.

There were significant differences in ^14^C-glyphosate translocation between the GR and GS populations, and there were even differences between the nine GR populations (Table 2). A greater movement of glyphosate to the rest of the plant was shown in the GS populations. At 96 HAT, 49 and 48% of ^14^C-glyphosate were taken up by the roots of Spain-1 and France C, respectively. On the contrary, the Line-3, Huerta, Alamo, and Sabiote populations presented lower movement of glyphosate to the roots, with not differences between them. The Bailen population showed the lowest amount of glyphosate in roots compared with all populations. The Wheat-B population exhibited the highest amount of glyphosate in shoot tissue, with 57%. The results obtained in the remaining populations were lower even in the GS populations, but the amount in roots was similar to the GR populations (Table 3).With the ^14^C-glyphosate visualization and amount in each population, the ^14^C-glyphosate distribution (Table 2, Fig. 5) could be exactly determined. The two greater GR populations, i.e., Line-3 and Huerta, revealed similar ^14^C-glyphosate amounts and distributions in the plant. These results suggested that glyphosate translocation is a mechanism involved in these nine GR populations due to the lower amount of glyphosate in roots.

**Table 3:** Parameter estimates of the equation used to calculate the sensitivity of EPSPS enzyme activity (I_50_) to glyphosate in extracts from leaf tissue of *Lolium rigidum* populations.

Population	d	b	I_50_ (µM)	(95% CI)		RI	*p-value*
				Upper	Lower		
Line-3	100.15	1.17	802.32	861.38	743.26	44.97	0.0001
Huerta	100.11	0.94	570.44	653.41	567.47	31.97	0.0001
Sabiote	100.22	0.86	439.35	458.29	420.41	24.62	0.0001
Bailen	100.01	1.37	327.21	332.77	321.65	18.34	0.0001
France-A	99.91	1.43	43.33	47.08	39.58	2.42	0.3089
Wheat-B	100.27	1.21	51.88	53.03	50.73	2.90	0.0951
Alamo	100.25	1.29	39.77	41.70	37.84	2.22	0.1582
France-D	100.80	0.98	39.83	43.33	36.33	2.23	0.1025
Wheat-A	101.05	0.98	29.98	33.52	26.44	1.68	0.2435
Spain-1	101.17	1.04	21.70	24.48	18.92	1.21	0.1863
France-C	101.47	1.03	17.84	20.81	14.87	—	—

CI values are the 95% confidence intervals (*n* = 6). RI (Resistance Indices) = I_50_ (Resistant)/I_50_ (Susceptible). d is the coefficient corresponding to the upper asymptote, b is the slope of the line.

### EPSPS enzyme activity

**Figure 6:**
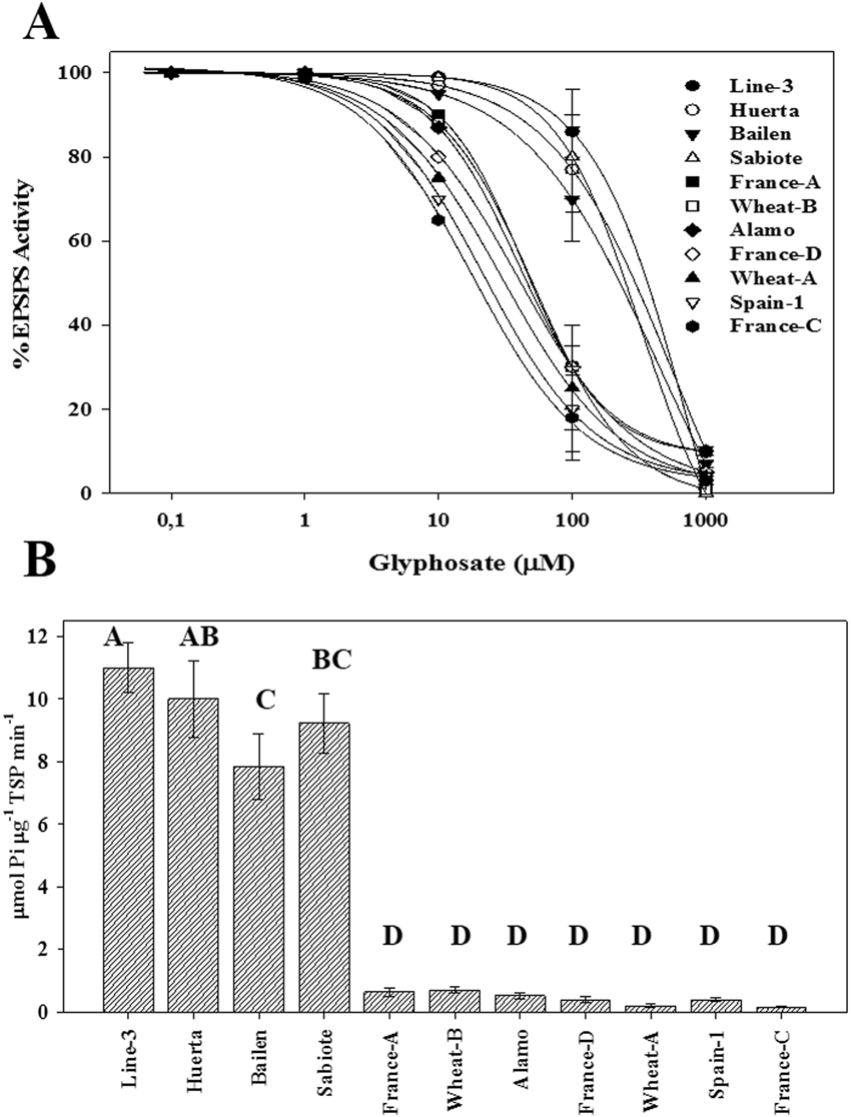
(**A**) EPSPS enzyme activity expressed as percentage of the untreated control in leaf extracts of *Lolium rigidum* populations. Vertical bars are ± standard errors of the mean (*n* = 3). (**B**) Basal EPSPS activity of *Lolium rigidum* populations. Vertical bars are ± standard errors of the mean (*n* = 3).

Figure 6 shows the EPSPS enzyme activity (I_50_) for the GR and GS populations. The Line-3, Huerta, Sabiote, and Bailen populations exhibited the highest levels of EPSPS enzyme activity, showing 44.97-, 31.97-, 24.62-, and 18.34-fold higher activity with respect to France-C (Table 3). The other populations showed lower I_50_ values closer to those of the S-populations, with non-significant differences. Moreover, there were non-significant differences in Line-3 (P = 0.4840), Huerta (P = 0.7958), and Sabiote (P = 0.7770) with respect to Bailen (Table 3, Fig. 6A).

The basal enzyme activity was 11.03-to 0.16 µmol µg^−1^ protein min^−1^ for the Line-3 and France-C populations, respectively (Fig. 6B). The remaining populations showed similar values. No significant differences were found in the six populations that had lower values, i.e., France-A, Wheat-B, Alamo, France-D, Wheat-B, and Spain-1, with respect to France-C.

### EPSPS gene sequencing

**Figure 7:**
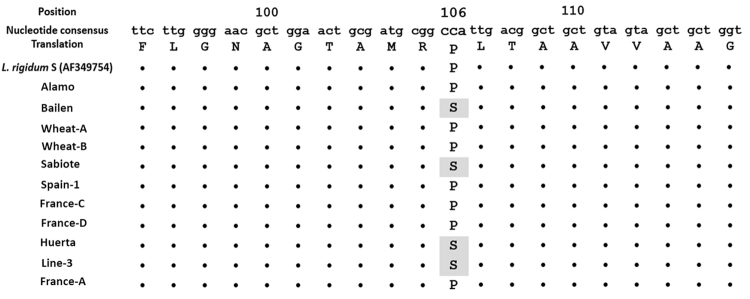
Partial protein sequence alignment of the EPSP synthase of *Lolium rigidum* populations. Five purified PCR products per population.

We sequenced 543 bp of the EPSPS gene of *L. rigidum* plants of the GR and GS populations. The fragments were aligned and numbered based on a published EPSPS sequence of *L. rigidum* (GenBank: AF349754.1). The partial EPSPS gene sequence in the Line-3, Huerta, Sabiote, and Bailen populations revealed a single nucleotide substitution of CCA to TCA at codon 106, which was an amino acid substitution from proline to serine (Fig. 7).

## Discussion

In Spain and France, chemical weed control in perennial crops has been extensively relied on glyphosate. Consequently, cases of glyphosate resistance to *L. rigidum* have already been reported in both countries^4,15,30,31^. Good uptake, excellent translocation to growing sites, nil or limited degradation and a slow mode of action are the primary reasons for the excellent efficacy of glyphosate^32^. Unfortunately, repeated use of glyphosate along with the absence of other proactive methods has greatly increased the risk of glyphosate resistance. Further reliance on glyphosate is likely since the number of approved herbicides in the EU is further reduced. It is noticeable that in 2009 after completion of the review process under Article 8 of Directive 91/414/EEC out of the 981 active ingredients approved for use in 1993 just 26% were approved, 67% were withdrawn and 7% were rejected^33^.

The resistance reported by Fernandez *et al*.^4,15,30,31^ was confirmed in a population from perennial crops. Moreover, in this study, GR cases among both annual and perennial crops have been reported. In Spain, *L. rigidum* has been reported as GR in perennial crops, but never before in annual crops^5^. In our study, the Wheat-B *L. rigidum* population had been exposed to glyphosate for several years after wheat harvest and before sowing of the next crop in order to control the weeds and prepare the soil surface for the spring crop, usually sunflower or corn. In summary, five to nine GR populations were originated from olive groves, three from vineyards, and one from a wheat field. Our results highlight the failure of the followed weed management strategy in terms of the emergence of herbicide resistance issues. In such cases, either the use of active ingredients with a different mode of action or the mechanical control could be beneficial for the farmers, as previously reported by Fernandez *et al*.^4^. Under that approach, the use of other herbicides like oxyfluorfen for pre-emergence and/or early post-emergence has been significantly increased^4,31^. Unfortunately, the use of oxyfluorfen to prevent and control GR weed populations has continued with no other chemical rotations, and then, oxyfluorfen effectiveness has declined, resulting in a case of multiple resistance^4^. Interestingly, cases of glyphosate resistance in other species of the *Lolium* genus have been also described, including *L. multiflorum* from Spain^14^, and *L. perenne* from Portugal^4^.

In the present study, both NTSR and TSR mechanisms were studied in order to collect the required information from all the range of mechanisms. In many studies, only one type of mechanism was studied, and usually the TSR mechanism^2,19^. To date, weeds with both mechanisms of resistance to herbicides represent the usual situation partially due to the continuous herbicide selection, frequency of genes related with the resistance and different environments^20^. The wide range of the values obtained from our different assays indicated two different resistance mechanisms (reduced glyphosate translocation [NTSR] and amino acid substitution [TSR]). Some populations exhibited both NTSR and TSR mechanisms^12^.

It has to be noted that some populations characterized as GS originated from fields that the farmers had previously marked as potentially GR (Supplementary Table S1), although screening under optimal conditions determined their susceptibility. Indeed, low efficacy cases are often due to applications at a higher growth stage (beginning of flowering stage), with the wrong pressure, and/or ignoring the environmental factors during the use of herbicides^30,34^. These situations could result in a possible resistance in a few years, but certainly they can not be considered to be GR cases^6,20^. The populations characterized as being GR could be initially due to these events and, numerous applications for successive years, to increases in the recommended field dose, without the use of herbicides with different mode of action^4,30,31^.

Our dose-response results based on the RI were similar to those of other studies on *L. rigidum*. For instance, Fernandez *et al*.^15^ reported a RI of 5.8, with TSR being the mechanism involved. Fernandez *et al*.^4^ also reported a RI of 21.4, where there was multiple-resistance to non-selective herbicides. Vila-Aiub *et al*.^34^ found a RI of 6–8 at different temperatures, and Adu-Yeboah *et al*.^35^ found an RI of 15–30, with NTSR being the mechanism involved, as in many other cases. The resistance definition might be a subjective concept, but it occurs when the dose-response values are higher than the recommended field dose, and are backed by the resistance mechanisms involved. RIs vary due to many reasons such as the higher or lower susceptibility of GS-plants used for comparison to the GR-plants, the different mechanisms involved that result in higher or lower RI value, and the existence of multiple- or cross-resistance.

The accumulation of shikimic acid reflects EPSPS inhibition caused by glyphosate application^10,36^. Thus, a higher accumulation results in higher toxicity of glyphosate in the S-plants, on the contrary to the R-plants, which show poor or no EPSPS inhibition^15^. Therefore, from the results obtained in this assay and the dose-response results, glyphosate resistance was confirmed in nine populations of *L. rigidum* (Figs 3 and 4, Table 1).

A reduction in ^14^C-glyphosate absorption, due to differences in cuticle properties, was previously reported in some R-weeds^23,27^. These GR weeds did not exhibit high levels of resistance due to the use of several commercial products with different formulations, which probably allowed a higher penetration. Our results demonstrate that different glyphosate absorption is not the mechanism involved in these nine GR populations. The nine GR populations showed reduced ^14^C-glyphosate translocation from the treated leaf to the rest of the plant (Fig. 5). Moreover, this is probably the first resistance mechanism that allows the plants to defend themselves against the herbicide. However, it remains unclear which mechanism develops first, i.e., NTSR or TSR^2^. Glyphosate is a systemic herbicide and must reach the enzymatic sites of root and shoot meristems in order to act. Any reduction in its translocation to these sensitive sites has a negative effect on its efficacy^14^. Similar results in ^14^C-glyphosate translocation were reported in GR *L. rigidum* plants^28,30,37^, and other GR species^10,23,31,38,39^. In the present study it is given the first evidence of the different resistance levels. In particular, the resistance in the Wheat-B, France-D, Alamo, France-A, and Wheat-A populations is due to the reduced glyphosate translocation. These populations involve only one mechanism of herbicide resistance, but the remaining higher R-populations, which also have reduced glyphosate translocation seem to involve both mechanisms, NTSR and TSR. The results obtained in the dose-response assays, with the different levels of resistance (Table 1) support this affirmation. The existence of both mechanisms in the same population results in high resistance levels. This could be because the first mechanism, which was probably reduced glyphosate translocation, was followed by the development of a second mechanism, such as amino acid substitution, with the repeated use of glyphosate, leading to higher resistance levels. Moreover, the Wheat-B population showed a unique translocation pattern, exhibiting the highest amount of glyphosate in shoot tissue, up to 57% (Table 2). This result might lead to the speculation that vacuolar sequestration or metabolism may be involved, i.e., another NTSR mechanism involved. However, vacuolar sequestration is a mechanism that has not been adequately studied; and glyphosate metabolism does not seem to be a frequent resistance mechanism^8^ with only a few species able to transform glyphosate into non toxic compounds^20,26^.

As evidenced by our results and those obtained in other studies, the TSR mechanism is associated with a smaller amount of shikimic acid and higher levels of EPSPS activity^10,40,41^. Amino acid substitutions at position 106 of the EPSPS protein have been reported in several weeds, such as *Echinochloa colona* ^16^, *Amaranthus tuberculatus* ^42^, *D. insularis* ^26^, *E. indica* ^13,43^, *L. rigidum* ^15,44^, *L. multiflorum* ^14^, and *L. perenne* ^28^. Mutations at this position generally provide low levels of resistance to glyphosate, with approximately 2–6-fold resistance level^12,45^. However, a double mutation at Pro-106 and Thr-102 found in *E. indica* and *Bidens pilosa* may result in a higher resistance^10,11,46^. The Line-3, Huerta, Sabiote, and Bailen populations showed Pro-106 mutations, and they had higher levels of resistance (>10-fold), which was due to an additional NTSR mechanism (reduced glyphosate translocation). Therefore, these populations (Line-3, Huerta,Sabiote, and Bailen) were characterized as being GR with NTSR and TSR mechanisms involved. The presence of the latter in these populations makes it difficult to determine the contribution of each mechanism^20,47^. For example, Wheat-B and France-D had the NTSR mechanism, exhibiting similar resistance levels to Bailen, in which these two mechanisms were involved. This situation, with both mechanisms was reported in *E. colona* ^16^, *Parthenium hysterophorus* ^20^, *L. rigidum* ^29^, and *A. tuberculatus* ^47^. Accumulation of these mechanisms could be due to cross-pollination, selection by glyphosate, and environmental conditions^12,20,34^.

In summary, all GR *L. rigidum* populations exhibited a non-target-site mechanism with reduced translocation. Moreover, four populations had target-site alterations, with Pro-106-Ser substitution in the EPSPS protein. The occurrence of both resistance mechanisms results in higher resistance levels. Several populations and their dispersal into new areas should be further studied in order to evaluate their resistance in the field crops. This action could help farmers; control not only glyphosate-resistance but also multiple-resistance in weeds of high agronomic importance^31^. Our results would help in establishing a long-term management strategy against herbicide resistance in *Lolium* spp. As previously stated by Powles^48^, a major lesson evident from more than three decades of glyphosate use to control billions of plants worldwide is that, where diversity in weed management systems is maintained, weed control by glyphosate can be sustainable. Increased awareness of weed resistance by farmers, extended field monitoring, understanding of the involved mechanisms and integrated weed management strategies are crucial to delay or prevent resistance.

## Material and Methods

### Plant material

Mature seeds were collected in July 2015 from different fields of southern Spain and France. Some farmers told us the glyphosate weed control failed in their crops. Then, we collected all these cases, and moreover, we collected seeds from nearby areas to obtain more information. We obtained the historical field application records for only some populations out of the 45 populations. Forty-five populations were collected and characterized by glyphosate application assays as being resistant (GR), or susceptible (GS) to glyphosate. Around 20 plants with mature seeds were randomly collected in 50 m^2^ from the coordinate GPS of each population (Supplementary Table S1). Then, they were cleaned and arranged for germination.

All the mature seeds were germinated in Petri dishes with filter paper moistened with distilled water and they were placed in a growth chamber at 28/18 °C (day/night) with a photoperiod of 16 h, 850 µmol m^−2^ s^−1^ photosynthetic photon flux, and 80% relative humidity. The populations were transplanted into pots containing sand/ peat in a 1:2 (*v/v*) ratio and placed in a greenhouse at 28/18 °C (day/night) with a 16 h photoperiod.

### Screening of populations

Fifty plants from each population were sprayed with 1080 g ae ha^−1^ (3 L ha^−1^ [recommended field dose]) of glyphosate (Roundup®, 360 g ae L^−1^ as isopropylamine salt, Monsanto, Spain). Glyphosate was applied at the 3–4 leaf growth stage with a laboratory chamber sprayer (SBS-060 De Vries Manufacturing, Hollandale, MN) equipped with 8002 flat fan nozzles delivering 200 L ha^−1^, at 250 kPa at the height of 50 cm, and at room temperature.

A visual evaluation of damage ranging from a scale of 0 to 100 was performed to determine the glyphosate resistance at 21 days after application (DAT). A score of 100 meant total glyphosate control, and 0 meant no control. Populations with a score of 85 or over were characterized as being GS, while populations with a lower score were characterized as being GR (Fig. 1). The glyphosate application and evaluation were repeated twice. We observed the mortality or survival plants and damage of chlorotic in leaves in all populations. In the following assays, we studied all GR populations and two GS populations (one from Spain [Spain-1] and another from France [France-C]). The reason for using these two GS populations was because no differences were observed among the GS populations studied, showing total control without any regrowth. Therefore, we selected a population from Spain and another from France.

### *Lolium* weed species characterization using molecular markers

In this research, AFLP markers were used as a system for *Lolium* species characterization following the methodology described by Fernandez *et al*.^4^ and Ma *et al*.^49^. The plant material used was two GS populations (Spain-1, and France-C), and nine GR ones (Alamo, Bailen, Wheat-A, Wheat-B, Sabiote, France-A, France-D, Huerta, Line-3). Twelve plants of each putative *L. rigidum* were used for molecular analysis. Additionally, twelve reference susceptible plants (*L. multiflorum* and *L. perenne*) were included in the study.

DNA was extracted from the leaf tissue (50 mg), using the Speedtools DNA Extraction Plant kit (BIOTOOLS, Madrid, Spain). The quality and concentration of the DNA was evaluated by spectrophotometer analysis with 260 nM and 280 nM light absorption. AFLP analysis was carried out using the fluorescent AFLP IRDye kit for Large Plant Genome Analysis (LI-COR Biosciences). Template preparation was performed following the protocol included in the kit, including digestions with *Eco*RI and *Mse*I restriction enzymes (Invitrogen). The primers for selective amplification are described in Fernandez *et al*.^4^.

AFLP products were separated by polyacrylamide electrophoresis by using an automated sequencer (LICOR 4300). Polymorphic AFLP markers and primers were identified and individuals were scored for presence or absence of AFLP fragments, using the computer package SAGAMX 2 GENERATION. UPGMA analysis was performed with AFLP marker data using the computer program NTSYSpc 2.2.

### Dose-Response assays

Herbicide treatments were applied at the 3–4 leaf growth stage of *L. rigidum* populations. Glyphosate applications were applied with a laboratory chamber sprayer under the same conditions described previously. The following glyphosate (Roundup®, 360 g ae L^−1^ as isopropylamine salt) rates were used: 0, 62.50, 125, 250, 500, 1000, 2000, 4000, and 8000 g ae ha^−1^. The experiment was arranged using ten replicates per rate and was repeated twice. Plants were cut at the soil surface 21 DAT, and fresh weight reduction (GR_50_) and survival (LD_50_) at 50% were measured.

### Shikimic accumulation assay

Leaf segments (5 mm diameter) were harvested from the youngest fully expanded leaf from a pool of 15 plants per population at the 3–4 leaf growth stage^50,51^. Approximately 50 mg of fresh tissue was transferred to 2 mL eppendorf tubes containing 1 mL of 1 mM NH_4_H_2_PO_4_ (pH 4.4). Glyphosate was added to eppendorfs at following concentrations: 0, 0.1, 0.5, 1, 5, 10, 50, 100, 200, 400, 500, 600, and 1000 µM. The eppendorfs were incubated in a growth chamber during 24 h under the above conditions. After 24 h, the eppendorfsd were stored at −20 °C until their analysis. They were then removed from the freezer and thawed at 60 °C for 30 min. 250 µL of 1.25 N HCL was added to each tube. Again, they were left at 60 °C for 15 min. A 125 µL aliquot from each tube was pipetted into a new 2 mL eppendorf, and 500 µL of periodic acid and sodium metaperiodate (0.25% [wt/v] each) was added. They were incubated at room temperature for 90 min, after which 500 µL of 0.6 N sodium hydroxide and 0.22 M sodium sulfite was added. All the eppendorf tube contents were then transferred to glass vials. Samples were measured in a spectrophotometer at 380 nm within 30 min. Each glyphosate concentration contained six replicate plants of each population. The assay was repeated twice. The results were expressed as µg shikimic acid µg^−1^ fresh weight.

### ^14^C-glyphosate absorption and translocation

The assays were carried out according to methodology described by Fernandez-Moreno *et al*.^30,51^. ^14^C-glyphosate (American Radiolabeled Chemicals, Inc., Saint Louis, MO, USA) was added to commercial glyphosate with a specific activity of 0.834 KBq µ^−1^. The final glyphosate concentration corresponded to 300 g ae ha^−1^ applied in 200 L ha^−1^. Plants at the 3–4 leaf growth stage were treated with a drop of 1 µL (0.834 KBq plant^−1^) with a micropipette (LabMate Soft, HTL Lab Solutions, Warsaw, Poland) onto the adaxial surface of the second leaf. The treated leaf was washed with 3 mL of water: acetone (1:1 v/v) solution to remove the non- ^14^C-glyphosate absorption at 96 h after treatment (HAT). The rinsate was mixed with 2 mL of scintillation cocktail and analyzed by liquid scintillation spectrometry (LSS) on a scintillation counter (Beckman LS 6500, Fullerton, CA, USA). The remainder of the plant was carefully removed from the pot, and its roots were gently washed with distilled water. The plant was divided into treated leaf, remaining shoot tissue, and roots. The plant parts thus obtained were dried at 60 °C for 96 h and combusted in a Packard Tri Carb 307 biological sample oxidizer (Packard Instruments, Meriden, USA). Evolved ^14^CO_2_ was trapped and counted by LSS in a 18-mL mixture of Carbo-Sorb E and Permafluor E + (1:1 v/v) (Perkin-Elmer, Packard Bioscience BV). The amount of radiolabel deposited was checked by washing a treated leaf excised immediately after deposition. There were five replicates, and the experiment was arranged in a completely randomized design. The assays were repeated twice. The proportion of absorbed herbicide was expressed as [kBq in combusted tissue/(kBq in combusted tissue + kBq in leaf washes)] × 100.

### ^14^C-glyphosate visualization

^14^C-glyphosate translocation was visualized using a phosphor imager (Cyclone, Perkin-Elmer). Plants were treated and collected in the same way described in the absorption and translocation assays. The whole plants were gently rinsed, pressed, and then left to dry at room temperature during four days. Next, the dried plants were placed adjacent to a 25 cm × 12.5 cm phosphor storage film for 13 h and scanned for radiolabel distribution on a phosphor imager. The experiment was carried out with three plants per population.

### EPSPS enzyme activity assays

Samples of five g of leaf tissue (3–4 leaf growth stage) from each population were ground to fine powder using a pestle in a mortar. The methodology by Sammons *et al*.^52^ was used for EPSPS extraction. The total content of proteins in the extract was measured according to the method of Bradford^53^ using a Kit for Protein Determination (Sigma-Aldrich, Madrid, Spain). The specific EPSPS activity in plants from each population was studied in the presence and absence (basal activity) of glyphosate. The EPSPS activity was determined using a EnzChek Phosphate Assay Kit (Invitrogen, Carlsbad, CA, USA). The glyphosate concentrations used were: 0, 0.1, 1, 10, 100, and 1000 µM. Three replicates at each glyphosate concentration were used, and the experiment was repeated three times. The release of phosphate on the bottom level was measured during 10 minutes at 360 nm in a spectrophotometer (DU-640, Beckman Coulter Inc. Fullerton, USA).

### EPSPS gene sequencing

EPSPS gene sequencing was conducted according to the protocol described by Alcantara-de la Cruz *et al*.^10^. Total RNA was isolated from leaves using TRIzol reagent (Invitrogen, Carlsbad, CA, USA) following the manufacturer’s instructions. RNA was then treated with TURBO DNase (RNase-Free; Ambion, Warrington, UK) to eliminate any DNA contamination and stored at −80 °C. cDNA synthesis was carried out from 2 μg of total RNA using a M-MLV (Moloney Murine Leukemia Virus) Reverse Transcriptase (Invitrogen, Carlsbad, CA, USA) in combination with oligo (dT)_12-18_ and random non namers (Amersham Biosciences, Amersham, UK) according to the manufacturer’s instructions. To amplify the EPSPS gene, primers previously designed by Perez-Jones *et al*.^54^ (forward: 5′ AGCTGTAGTCGTTGGCTGTG 3′; reverse: 5′ GCCAAGAAATAGCTCGCACT 3′) were used. The PCR reactions were carried out using cDNA from 50 ng of total RNA, 1.5 mM MgCl2, 0.2 mMdNTP, 0.2 μM of each primer, 1 × buffer, and 0.625 units of a 100:1 enzyme mixture of nonproofreading (*Thermus thermophilus*) and proofreading (*Pyrococcus furiosus*) polymerases (BIOTOOLS, Madrid, Spain) in a final volume of 25 μL. All PCR reactions were made in duplicate and cycling conditions were: 94 °C 3 min, 35 cycles of 94 °C 30 s, 55 °C 30 s and 72 °C 1 min; and a final extension cycle of 72 °C 10 min. An aliquot of the PCR product was loaded in a 1% agarose gel to check the correct band amplification. The rest of the PCR product was then purified using ExoSAP-IT® for PCR Product Clean-Up (USB, Ohio, USA) as indicated by the manufacturers. Five purified PCR products per population were sequenced (STAB VIDA, Caparica, Portugal).

### Statistical analysis

Dose-response and EPSPS enzyme activity data were subjected to non-linear regression analysis using a three-parameter log-logistic equation (Equation 1) to determine the glyphosate dose causing 50% reduction in growth (GR_50_), 50% mortality (LD_50_), or the herbicide rate 50% inhibition of EPSPS activity (I_50_).

1 $$y=([(d)/1+{(x/g)}^{b}]),$$

where *y* is the above ground fresh weight, the survival, or the enzyme activity expressed as the percentage of the non-treated control, *d* is the coefficient corresponding to the upper asymptote, *b* is the slope of the line, *g* is the GR_50_, LD_50_, I_50_, and *x* (independent variable) is the herbicide rate.

Regression analyses were conducted using the *drc* package^55^ for the statistical environment R^56^. Resistance indices (R/S) were computed as R-to-S GR_50_, LD_50_ or I_50_ ratios. To test for a common GR_50_, LD_50_, or I_50_ for R- and S-populations, i.e. Resistance Index equal to 1, a lack-of-fit test was used to compare the model consisting of curves with population-specific *g* values with a reduced model with common *g* ^55^.

Analysis of variance (ANOVA) was conducted to test for differences between GR and GS populations in the different assays. When needed, differences between means were separated using the Tukey HSD test at P < 0.05. Model assumptions of normal distribution of errors and homogeneous variance were graphically inspected. ANOVAs were conducted using the Statistix (version. 9.0) (Analytical Software, USA) software.

## Electronic supplementary material

Supplementary materials
