# Supplementary material for: Different levels of glyphosate-resistant Lolium rigidum L. among major crops in southern Spain and France
Source: Sci Rep. 2017 Oct 13;7:13116. doi: 10.1038/s41598-017-13384-2 (PMC5640615; doi:10.1038/s41598-017-13384-2)
Supplement: Supplementary file 1 — Supplementary materials [file 41598_2017_13384_MOESM1_ESM.docx]

**Supplementary Table S1.** Populations collected from different areas of Spain and France.

| Population | Name ^a^ | Coordinate GPS | Crop | Characterization^a^ |
| --- | --- | --- | --- | --- |
| 1 | Road | 38°01'40.3"N 4°04'41.1"W | Cotton | Susceptible |
| 2 | **Huerta** | 37°36'48.82"N 4°17'46.66"W | Vineyard | **Resistant** |
| 3 | OX1 | 39°58'47.2"N 0°10'17.0"W | Citrus | Susceptible |
| 4 | OX2 | 39°58'44.2"N 0°09'59.4"W | Citrus | Susceptible |
| 5 | AR | 38°01'31.5"N 4°04'24.3"W | Cotton | Susceptible |
| 6 | Villa | 8°01'05.1"N 4°05'00.1"W | Olive grove | Susceptible |
| 7 | **Alamo** | 38°17'43.3"N 2°56'6.25"W | Olive grove | **Resistant** |
| 8 | Line-1 | 38°17'45.3"N 2°57'06.3"W | Olive grove | Susceptible |
| 9 | Line-2 | 38°17'42.1"N 2°56'56.3"W | Olive grove | Susceptible |
| 10 | **Line-3** | 38°17' 49.32"N 2°57' 7.28"W | Olive grove | **Resistant** |
| 11 | Line-4 | 38°17'49.2"N 2°56'40.5"W | Olive grove | Susceptible |
| 12 | Parcela | 36°41'02.6"N 4°35'18.9"W | Citrus | Susceptible |
| 13 | Pipas-1 | 37°47'34.5"N 4°54'58.3"W | Sunflower | Susceptible |
| 14 | Pipas-2 | 37°47'16.9"N 4°54'59.7"W | Sunflower | Susceptible |
| 15 | Nadx | 37°46'36.6"N 4°55'32.7"W | Fallow | Susceptible |
| 16 | **Bailen** | 38º10´00”N 3º42´39.36”W | Olive grove | **Resistant** |
| 17 | ABX | 39°57'37.1"N 0°10'12.9"W | Citrus | Susceptible |
| 18 | Anton | 38°08'26.2"N 3°06'39.3"W | Olive grove | Susceptible |
| 19 | Pilar | 38°08'06.5"N 3°05'32.7"W | Olive grove | Susceptible |
| 20 | **Sabiote** | 38°03'29.9"N 3°19'6.9"W | Olive grove | **Resistant** |
| 21 | Depu | 38°06'50.8"N 3°06'37.1"W | Olive grove | Susceptible |
| 22 | Polid | 38°07'26.4"N 3°04'03.5"W | Olive grove | Susceptible |
| 23 | Corn-A | 37°47'44.9"N 4°59'47.1"W | Corn | Susceptible |
| 24 | Corn-B | 38°00'19.7"N 4°10'46.5"W | Corn | Susceptible |
| 25 | **Wheat-A** | 38°5'29.21"N 3°9'53.95"W | Olive grove | **Resistant** |
| 26 | **Wheat-B** | 38°5' 33.05"N 3° 9'41.30"W | Wheat | **Resistant** |
| 27 | Wheat-C | 37°55'37.2"N 4°41'40.8"W | Wheat | Susceptible |
| 28 | Wheat-D | 37°55'36.0"N 4°41'01.3"W | Wheat | Susceptible |
| 29 | Antex | 37°50'21.2"N 4°49'25.0"W | Olive grove | Susceptible |
| 30 | Montain | 38°06'39.7"N 3°02'33.2"W | Olive grove | Susceptible |
| 31 | Jaen | 37°58'50.2"N 3°48'37.7"W | Olive grove | Susceptible |
| 32 | **France-A** | 44°06'41.0"N 4°34'53.2"E | Vineyard | **Resistant** |
| 33 | **France-C** | 44°07'55.7"N 4°41'53.3"E | Vineyard | **Susceptible** |
| 34 | **France-D** | 44°05'38.7"N 4°38'37.3"E | Vineyard | **Resistant** |
| 35 | France-E | 46°51'3.85"N 0°59'7.13"W | Wheat | Susceptible |
| 36 | France-F | 43°57'33.35"N 1°19'41.00"E | Olive grove | Susceptible |
| 37 | France-G | 43°57'38.92"N 1° 9'37.51"E | Wheat | Susceptible |
| 38 | France-H | 44° 0'34.78"N 1° 4'2.50"E | Olive grove | Susceptible |
| 39 | **Spain-1** | 38°07'17.3"N 3°05'44.7"W | Olive grove | **Susceptible** |
| 40 | Spain-2 | 38°08'01.4"N 3°05'30.2"W | Olive grove | Susceptible |
| 41 | PPO | 38°08'23.4"N 3°03'13.6"W | Olive grove | Susceptible |
| 42 | ZR | 37°50'28.1"N 4°49'47.4"W | Sunflower | Susceptible |
| 43 | Spain-Z | 37°31'38.0"N 4°39'25.4"W | Vineyard | Susceptible |
| 44 | Cordoba | 37°49'29.2"N 4°59'40.2"W | Citrus | Susceptible |
| 45 | Almodob | 37°49'42.8"N 4°59'14.8"W | Citrus | Susceptible |

^a^ Names in bold are the populations chosen to study.
